# Supplementary material for: HLA-F and LILRB1 Genetic Polymorphisms Associated with Alloimmunisation in Sickle Cell Disease
Source: Int J Mol Sci. 2023 Sep 2;24(17):13591. doi: 10.3390/ijms241713591 (PMC10487752; doi:10.3390/ijms241713591)
Supplement: Supplementary file 1 [file ijms-24-13591-s001.zip › ijms-2552264-supplementary.pdf]

Supplementary Tables

	All patients	Non-alloimmunized patients	Alloimmunized patients	p-value (Chi 2)
HLA-G				
01:01:01	26/70 (37.1%)	15/38 (39.5%)	7/18 (38.9%)	0.97
01:01:02	14/70 (20.0%)	7/38 (18.4%)	2/18 (11.1%)	0.49
01:04:01	12/70 (17.1%)	8/38 (21.0%)	3/18 (16.7%)	0.70
01:04:04	3/70 (4.3%)	1/38 (2.6%)	1/18 (5.5%)	0.58
01:03:01	5/70 (7.1%)	1/38 (2.6%)	2/18 (11.1%)	0.19
01:05N	4/70 (5.7%)	1/38 (2.6%)	2/18 (11.1%)	0.19
others	6/70 (8.6%)	5/38 (13.2%)	1/18 (5.5%)	0.39
HLA-E				
01:01:01	30/70 (42.8%)	18/38 (47.4%)	6/18 (33.3%)	0.32
01:03:01	18/70 (25.7%)	11/38 (28.9%)	7/18 (38.9%)	0.45
01:03:02	18/70 (25.7%)	9/38 (23.7%)	5/18 (27.8%)	0.74
HLA-F				
01:01:01	54/70 (77.1 %)	29/38 (76.3%)	16/18 (88.9%)	0.27
01:01:02	12/70 (17.1%)	7/38 (18.4%)	1/18 (5.5%)	0.20
01:03:01	4/70 (5.7%)	2/38 (5.3%)	1/18 (5.5%)	0.96
rs2523393-A	42/70 (60.0%)	25/40 (62.5%)	7/16 (43.7)	0.20
rs1362126-C	38/60 (63.3%)	21/32 (65.6%)	7/14 (50.0%)	0.32
rs2523405-T	35/70 (50.0%)	21/40 (52.5%)	5/16 (31.2%)	0.15
KIR3DS1 presence	10/36 (27.8%)	14/21 (66.7%)	6/9 (66.7%)	1
NKG2C deletion	23/72 (31.9%)	13/42 (30.9%)	5/18 (27.8%)	0.81
LILRB1				
rs3760860-A	42/72 (58.3%)	20/42 (47.6%)	13/16 (81.2%)	0.02
rs3760861-A	42/72 (58.3%)	20/42 (47.6%)	13/16 (81.2%)	0.02

LILRB2				
rs383369-A	46/50 (92.0%)	27/28 (96.4%)	9/12 (75.0%)	0.04

Table S1. Allelic frequencies of the genes HLA Ib, KIR3DS1 presence, NKG2C deletion and LILRB1 and LILRB2 polymorphisms in all patients understudy and according to alloimmunization in patients who received blood products. Number of alleles out of total alleles are given, as well as allelic frequencies (in brackets). P-values comparing non-alloimmunized vs. alloimmunized patients are given.

	Non-alloimmunized patients	Alloimmunized patients	p-value (Chi 2)
RHD	21	9	
RHD*01/*01	15 (71.4%)	8 (88.9%)	0.09
RHD*01/*03N.01	1 (4.8%)	0 (0%)	0.53
RHD*01/*08N.01	1 (4.8%)	0 (0%)	0.53
RHD*01/*10.03	2 (9.5%)	1 (11.1%)	0.81
RHD*01/*10.05 or *05.01	1 (4.8%)	0 (0%)	0.53
RHD*03N.01/*03N.01	1 (4.8%)	0 (0%)	0.53
RHCE	21	9	
RHCE*01/*01	0 (0%)	2 (22.2%)	0.03
RHCE*01/*02	6 (28.6%)	1 (11.1%)	0.30
RHCE*01/*01.01	1 (4.8%)	2 (22.2%)	0.14
RHCE*01/*01.20.01	3 (14.3%)	2 (22.2%)	0.59
RHCE*01/*01.20.03	1 (4.8%)	0 (0%)	0.50
RHCE*01.01/*01.20.03	1 (4.8%)	0 (0%)	0.50
RHCE*01.01/*02	1 (4.8%)	0 (0%)	0.50
RHCE*01.01/*03	0 (0%)	1 (11.1%)	0.12
RHCE*01.20.01/*01.20.03	1 (4.8%)	0 (0%)	0.50

RHCE*01.20.01/*02	1 (4.8%)	0 (0%)	0.50
RHCE*01.20.01/*03	0 (0%)	1 (11.1%)	0.12
RHCE*01/*03	1 (4.8%)	0 (0%)	0.50
RHCE*02/*02	1 (4.8%)	0 (0%)	0.50
RHCE*02/*03 or *01/*04	4 (19.0%)	0 (0%)	0.16

Table S2. RHD and RHCE genotype according according to alloimmunization in SCD patients who received blood products. Number of genotypes out of total genotypes are given as well as their frequencies (in brackets). P-values comparing non-alloimmunized vs. alloimmunized patients are given.
